# Health outcomes among HIV-positive Latinos initiating antiretroviral therapy in North America versus Central and South America

**DOI:** 10.7448/IAS.19.1.20684

**Published:** 2016-03-18

**Authors:** Carina Cesar, John R Koethe, Mark J Giganti, Peter Rebeiro, Keri N Althoff, Sonia Napravnik, Angel Mayor, Beatriz Grinsztejn, Marcelo Wolff, Denis Padgett, Juan Sierra-Madero, Eduardo Gotuzzo, Timothy R Sterling, James Willig, Julie Levison, Mari Kitahata, Maria C Rodriguez-Barradas, Richard D Moore, Catherine McGowan, Bryan E Shepherd, Pedro Cahn, for the Caribbean, Central and South America Network for HIV epidemiology (CCASAnet) and the North American AIDS Cohort Collaboration on Research and Design (NA-ACCORD)

**Affiliations:** 1 Investigaciones Clínicas, Fundación Huésped, Buenos Aires, Argentina; 2 Division of Infectious Diseases, Vanderbilt University School of Medicine, Nashville, TN, USA; 3 Department of Biostatistics, Vanderbilt University School of Medicine, Nashville, TN, USA; 4 Department of Epidemiology, Johns Hopkins Bloomberg School of Public Health, Baltimore, MD, USA; 5 Department of Medicine, University of North Carolina, Chapel Hill, NC, USA; 6 Department of Internal Medicine, Universidad Central del Caribe, Bayamón, Puerto Rico; 7 Instituto de Pesquisa Clinica Evandro Chagas, Fundação Oswaldo Cruz, Rio de Janeiro, Brazil; 8 Fundación Arriarán and Facultad de Medicina, Universidad de Chile, Santiago, Chile; 9 Instituto Hondureño de Seguridad Social and Hospital Escuela Universitario, Tegucigalpa, Honduras; 10 Instituto Nacional de Ciencias Médicas y Nutrición, Mexico City, Mexico; 11 Instituto de Medicina Tropical Alexander von Humboldt, Lima, Peru; 12 Division of Infectious Diseases, University of Alabama at Birmingham, Birmingham, AL, USA; 13 Division of General Internal Medicine, Massachusetts General Hospital, and Harvard Medical School, Boston, MA, USA; 14 Division of Infectious Diseases, University of Washington, Seattle, WA, USA; 15 Infectious Diseases Section, Michael E. De Bakey VAMC, Baylor College of Medicine, Houston, TX, USA; 16 Department of Medicine, Johns Hopkins University, Baltimore, MD, USA

**Keywords:** HIV, antiretroviral therapy, highly active, mortality, Latin America, North America, cohort studies

## Abstract

**Introduction:**

Latinos living with HIV in the Americas share a common ethnic and cultural heritage. In North America, Latinos have a relatively high rate of new HIV infections but lower rates of engagement at all stages of the care continuum, whereas in Latin America antiretroviral therapy (ART) services continue to expand to meet treatment needs. In this analysis, we compare HIV treatment outcomes between Latinos receiving ART in North America versus Latin America.

**Methods:**

HIV-positive adults initiating ART at Caribbean, Central and South America Network for HIV (CCASAnet) sites were compared to Latino patients (based on country of origin or ethnic identity) starting treatment at North American AIDS Cohort Collaboration on Research and Design (NA-ACCORD) sites in the United States and Canada between 2000 and 2011. Cox proportional hazards models compared mortality, treatment interruption, antiretroviral regimen change, virologic failure and loss to follow-up between cohorts.

**Results:**

The study included 8400 CCASAnet and 2786 NA-ACCORD patients initiating ART. CCASAnet patients were younger (median 35 vs. 37 years), more likely to be female (27% vs. 20%) and had lower nadir CD4 count (median 148 vs. 195 cells/µL, *p*<0.001 for all). In multivariable analyses, CCASAnet patients had a higher risk of mortality after ART initiation (adjusted hazard ratio (AHR) 1.61; 95% confidence interval (CI): 1.32 to 1.96), particularly during the first year, but a lower hazard of treatment interruption (AHR: 0.46; 95% CI: 0.42 to 0.50), change to second-line ART (AHR: 0.56; 95% CI: 0.51 to 0.62) and virologic failure (AHR: 0.52; 95% CI: 0.48 to 0.57).

**Conclusions:**

HIV-positive Latinos initiating ART in Latin America have greater continuity of treatment but are at higher risk of death than Latinos in North America. Factors underlying these differences, such as HIV testing, linkage and access to care, warrant further investigation.

## Introduction

In 2012, the World Health Organization (WHO) estimated there were 1.3 million HIV-positive individuals in the United States and Canada and 1.5 million in the countries of Latin America (including Mexico) [1]. In the United States, over 16% of individuals self-identified as Hispanic or Latino in the 2010 US census (defined as persons of Cuban, Mexican, Puerto Rican, South or Central American or other Spanish culture or origin regardless of race). Latinos in the United States have a rate of new HIV infections approximately three times greater than among non-Hispanic whites, but lower rates of engagement at all stages of the care continuum [2–4]. US Latinos are also more likely to be diagnosed with HIV at later disease stages, and disparities in obtaining medical care can be exacerbated by a lack of insurance, linguistic barriers, stigma and differences in patient-provider communication [5–8].

For HIV-positive Latinos in Mexico, Central America and South America, the availability of HIV care and antiretroviral therapy (ART) has expanded rapidly over the past decade, with approximately 75% of the estimated 790,000 persons in need of ART able to access treatment [1]. Latin America has diverse economic and social contexts, and prior studies have found that mortality and programme retention vary considerably between countries [9–12]. A recent analysis of six Latin American countries and one Caribbean country found that the overall mortality rate after five years of ART, estimated to be 10%, was generally lower than that observed in sub-Saharan Africa, but higher than that in Europe and North America [12–16], although rates were heterogeneous between countries.

The goal of this study was to estimate regional differences in clinical characteristics at ART initiation, mortality, ART discontinuation or changes and virologic failure among HIV-positive Latinos in the Americas. We compared outcomes of HIV-positive Latino patients initiating ART in Canada and the United States (hereafter referred to as *North America*) to those in patients in six Latin American countries (Argentina, Brazil, Chile, Honduras, Mexico and Peru) using the regional databases of the International Epidemiologic Databases to Evaluate AIDS (IeDEA) network.

## Methods

### Participants and settings

Patient-level HIV treatment and care data were aggregated from two multinational regional consortia of IeDEA: the Caribbean, Central and South America Network for HIV research (CCASAnet) and the North American AIDS Cohort Collaboration on Research and Design (NA-ACCORD). These consortia have been profiled elsewhere [17,18].

Briefly, CCASAnet is a network of clinical sites in seven diverse Caribbean, Central and South American countries that pools data collected as part of HIV treatment and care programmes. In this study, we included data from eight CCASAnet sites in six countries: Hospital Fernández and Centro Médico Huésped in Buenos Aires, Argentina (HF/CMH-Argentina); Instituto Nacional de Infectologia Evandro Chagas in Rio de Janeiro, Brazil (INI-Brazil); Fundación Arriarán in Santiago, Chile (FA-Chile); Instituto Hondureño de Seguridad Social and Hospital de Especialidades in Tegucigalpa, Honduras (IHSS/HE-Honduras); Instituto Nacional de Ciencias Médicas y Nutrición Salvador Zubirán in Mexico City, Mexico (INNSZ-Mexico); and Instituto de Medicina Tropical Alexander von Humboldt in Lima, Peru (IMTAvH-Peru). The CCASAnet site in Haiti was not included, given Haiti's African ethnic ancestry and non-Hispanic cultural heritage.

**Figure 1:**
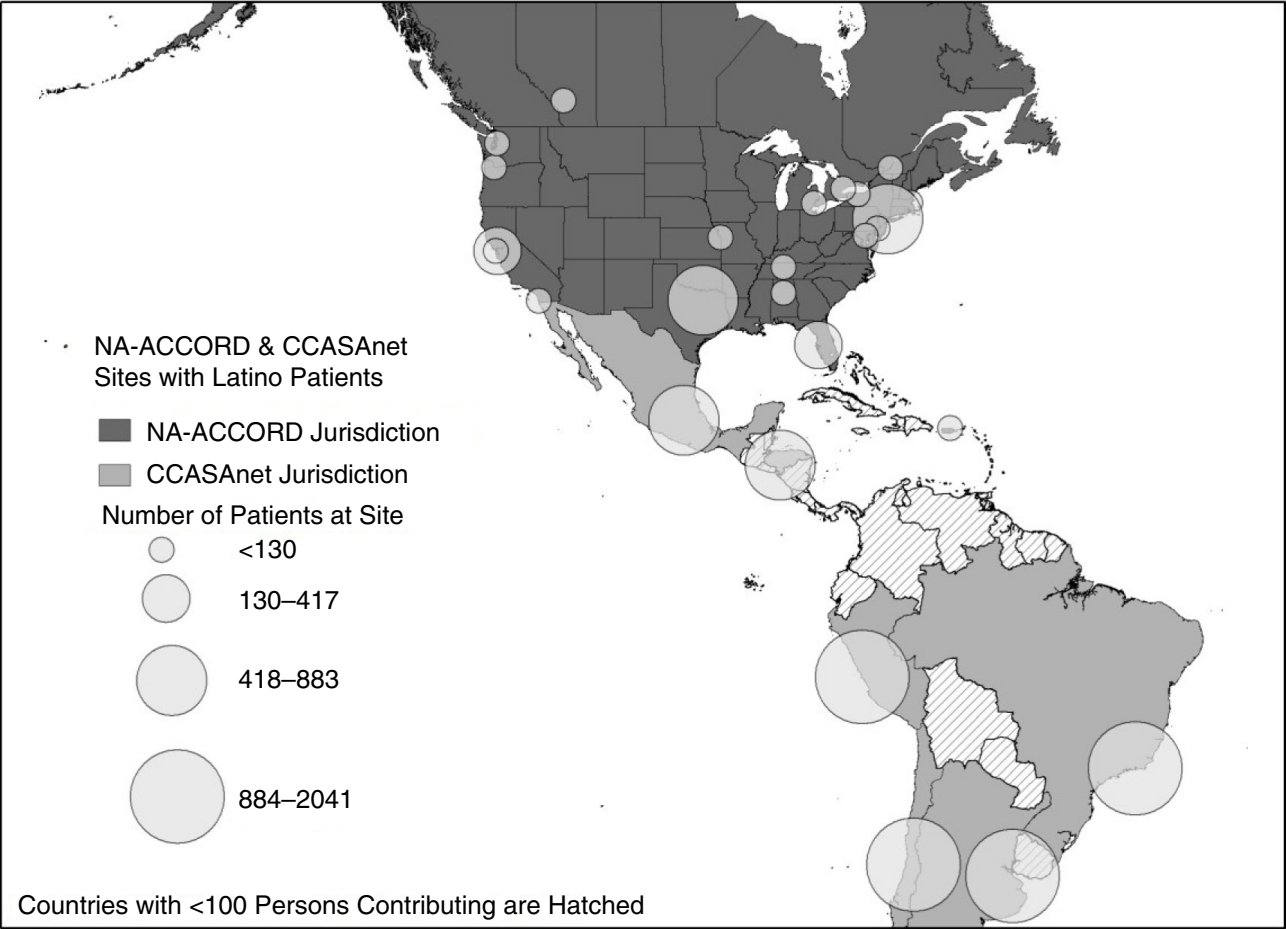
Geographic distribution of NA-ACCORD and CCASAnet patients included in the analysis cohort.

NA-ACCORD includes ≥200 sites in 25 single and multisite cohorts throughout the United States and Canada. In this study, data from 11 NA-ACCORD clinical cohorts were included: Fenway Community Health Center (Boston, MA), HIV Research Network, Johns Hopkins HIV Clinical Cohort (Baltimore, MD), Montreal Chest Institute Immunodeficiency Service Cohort, Ontario HIV Treatment Network Cohort Study, Retrovirus Research Center, Southern Alberta Clinic Cohort, Study of the Consequences of the Protease Inhibitor Era, University of Alabama at Birmingham 1917 Clinic Cohort, University of Washington HIV Cohort and Vanderbilt-Meharry Centers for AIDS Research (CFAR) Cohort. These sites included patients from 17 US states, Puerto Rico, and 4 Canadian provinces (Figure 1). Latinos in NA-ACCORD were identified based on either self-reported Hispanic ethnicity or self-reported country of birth in South America, Central America, Spanish-speaking countries in the Caribbean or Mexico. The NA-ACCORD cohorts not included in the analysis either did not have patients with Hispanic ethnicity or declined to provide data.

Our analysis cohort was restricted to ART-naive adult HIV-positive patients initiating ART between 2000 and 2011. Patients with an undetectable HIV-1 RNA viral load (VL) prior to reported date of ART initiation were excluded. Outcomes were assessed using data collected through 31 December 2011.

Clinical and epidemiological data were collected at each centre, de-identified and sent to the Data Coordinating Center at Vanderbilt University, USA. Institutional ethics review boards from all sites and Vanderbilt approved the project, waiving the requirement for individual patient informed consent.

### Outcomes

The time from ART initiation to each of five long-term outcomes was assessed: all-cause mortality, treatment interruption, major treatment change, virologic failure and loss to follow-up. ART was defined as a combination of three antiretroviral agents from at least two classes, or a triple nucleoside/nucleotide reverse transcriptase inhibitor regimen containing abacavir or tenofovir. Patients without an event were censored at 31 December 2011 or the date of their last clinical, pharmacy or laboratory visit, whichever occurred first.

Participant deaths in NA-ACCORD are recorded by the individual cohorts when the death can be confirmed, and deaths among patients lost to care are ascertained using routine surveillance and matching of US national and Canadian provincial death indexes. In CCASAnet, methods to ascertain death differ across sites. At IHSS/HE-Honduras, deaths are recorded after field workers contact patient family members following a missed visit. At all other sites, deaths occurring at affiliated hospitals or other clinical sites are recorded, as are notifications of deaths by relatives of patients. Additionally, study staff at INI-Brazil, FA-Chile, INNSZ-Mexico and IMTAvH-Peru sites query government death registry databases for patients lost to care at least annually.

Treatment interruption was defined as an interruption of the initial ART regimen for >14 days. Major treatment change included switching to a “second-line” regimen, defined as (1) a change to a boosted protease inhibitor (PI)-based regimen; (2) switching to a different PI if a patient's first regimen included a boosted PI; (3) switching to a “third-line” drug (darunavir, tipranavir, etravirine, enfuvirtide, maraviroc or raltegravir); or (4) changing to non-nucleoside reverse transcriptase inhibitor (NNRTI), boosted PI or switching, deleting or adding a third-line drug for those patients who had started treatment on a third-line drug. Virologic failure was defined as at least one of the following: (1) VL >400 copies/mL after six months of therapy, (2) VL <400 copies/mL followed by a single value >1000 copies/mL or (3) VL <400 copies/mL followed by two consecutive VL >400 copies/mL. The threshold of 400 copies/mL was chosen based on the detection limit for assays used at many of the sites during the study period. Patients who did not have a VL measurement after ART initiation were excluded from the virologic failure analyses. Patients who had a gap between VL measurements of greater than one year were censored at the last measurement prior to the gap. Loss to follow-up (LFU) was defined as lack of information about the patient within 180 days prior to the database closing date; for patients lost to follow-up, the date of LFU was defined as date of their last visit.

### Statistical analysis

Univariate comparisons were performed using Wilcoxon rank sum and chi-square tests. Kaplan-Meier estimates and corresponding confidence intervals (CIs) of the probability of mortality were calculated for each consortium. The cumulative incidence and corresponding 95% CI of treatment interruption, ART regimen change and virologic failure were also estimated and these models accounted for mortality as a competing risk. Separate multivariable Cox proportional hazards models were fit to compare the hazard of death, major treatment modification and treatment failure between patients in CCASAnet and NA-ACCORD. All multivariable models were adjusted for age, sex, reported mode of transmission, nadir CD4+ T-cell count (CD4 count) and clinical AIDS prior to treatment initiation, initial ART regimen and year of ART initiation. Restricted cubic splines were used for continuous variables to account for non-linear trends [19]. Multiple imputation was implemented to impute missing covariate values [20].

Different frequencies of VL measurement between cohorts may have biased the results since patients with more VL measurements had more opportunities to be classified as a treatment failure. We thus compared the incidence of a detectable VL greater than six months after ART initiation while accounting for the number of VL measurements using a multivariable negative binomial regression model. We report the adjusted relative proportions of detectable VL per measurement and corresponding 95% CI.

Brazilian HIV treatment sites contributing data to CCASAnet were included in the primary analysis, as were Brazilian patients living in the United States or Canada and enrolled in NA-ACCORD (the US census definition of *Latino* includes Brazilians). However, the ethnic heritage of Brazil includes a large component of African migration, and we performed subregion analyses which excluded patients initiating ART in Brazil and patients born in Brazil initiating ART in North America. We also performed a subregion analysis comparing NA-ACCORD patients reporting Mexico as their country of origin with CCASAnet patients from Mexico.

All analyses were performed using R version 3.1.2; analysis code is available at www.biostat.mc.vanderbilt.edu/ArchivedAnalyses.

## Results

From 2000 to 2011, a total of 11,186 ART-naive Latino patients initiated ART at one of 19 sites; 8400 (75%) at one of 8 CCASAnet sites in Latin America and 2786 (25%) in one of 11 NA-ACCORD cohorts with clinical sites in 17 US states and 4 Canadian provinces (Figure 1). CCASAnet patients included 2041 initiating ART in Argentina, 1792 in Brazil, 1347 in Chile, 882 in Honduras, 804 in Mexico and 1534 in Peru. Latinos initiating ART in North America included 294 with a known country of birth (156 from Mexico, 40 from other Central American countries, 69 from South American countries and 29 from the United States); the remaining 2492 patients self-reported as Latino.

Demographic and clinical characteristics of patients starting ART in each cohort are shown in Table 1. Patients initiating ART in CCASAnet were significantly younger (median 35 vs. 37 years old), more likely to be female (27% vs. 20%) and less likely to have acquired HIV through injection drug use (2% vs. 12%) than Latinos initiating ART in NA-ACCORD (*p*<0.001 for all). Median follow-up was 3.3 years (interquartile range (IQR): 1.2 to 6.0) for patients initiating ART in CCASAnet and 2.7 years (IQR: 1.0 to 5.6) for Latinos in NA-ACCORD.

**Table 1:** Description of cohort participants at antiretroviral therapy initiation

	CCASAnet (*n=*8400)	NA-ACCORD (*n=*2786)	*p*
Age, years	35 (29 to 43)	37 (30 to 44)	<0.001
Sex			<0.001
Female	2268 (27.0%)	550 (19.7%)	
Male	6132 (73.0%)	2236 (80.3%)	
Route of infection			<0.001
Heterosexual	3841 (45.7%)	1040 (37.3%)	
MSM	3248 (38.7%)	1313 (47.1%)	
IDU	179 (2.1%)	336 (12.0%)	
Other	63 (0.8%)	74 (2.7%)	
Unknown	1069 (12.7%)	23 (0.9%)	
Clinical stage			<0.001
AIDS	2443 (29.1%)	605 (21.7%)	
Not AIDS	4561 (54.3%)	1735 (62.3%)	
Missing	1396 (16.6%)	446 (16.0%)	
Nadir CD4 count, cells/µL	148 (54 to 246)	195 (68 to 298)	<0.001
Missing	980 (11.7%)	170 (6.1%)	
Baseline CD4 count, cells/µL	150 (55 to 253)	212 (79 to 323)	<0.001
Missing	1422 (16.9%)	248 (9%)	
Baseline viral load (log_10_)	5.0 (4.5 to 5.4)	4.8 (4.2 to 5.3)	<0.001
Missing	2708 (32.2%)	345 (12.4%)	
Initial regimen			<0.001
NNRTI	6699 (79.8%)	1240 (44.5%)	
Boosted PI	1183 (14.1%)	974 (35.0%)	
Other	518 (6.2%)	572 (20.5%)	
Nucleoside backbone			<0.001
ZDV+3TC	5604 (66.7%)	597 (21.4%)	
TDF+FTC	640 (7.6%)	1564 (56.1%)	
TDF+3TC	562 (6.7%)	127 (4.6%)	
D4T+3TC	873 (10.4%)	123 (4.4%)	
ABC+3TC	331 (3.9%)	183 (6.6%)	
Other	390 (4.6%)	192 (6.9%)	
Regimen anchor			<0.001
EFV	5470 (65.1%)	1145 (41.1%)	
NVP	1211 (14.4%)	83 (3.0%)	
NFV	78 (0.9%)	454 (16.3%)	
LPV+ritonavir	419 (5.0%)	316 (11.3%)	
ATV+ritonavir	279 (3.3%)	454 (16.3%)	
Other	943 (11.2%)	517 (18.6%)	
Initiation year	2007 (2004 to 2009)	2007 (2003 to 2009)	<0.001
VL measurements per person-year	1.95 (1.13 to 2.82)	3.38 (2.50 to 4.41)	<0.001
At least one VL measurement			<0.001
Yes	7522 (89.5%)	2683 (96.3%)	
No	878 (10.5%)	103 (3.7%)	

CCASAnet, Caribbean, Central and South America Network for HIV; IDU, injection drug use; MSM, men who have sex with men; NA-ACCORD, North American AIDS Cohort Collaboration on Research and Design; NNRTI, non-nucleoside reverse transcriptase inhibitor; PI, protease inhibitor; VL, viral load. Antiretroviral agents: 3TC, lamivudine; FTC, emtricitabine; ABC, abacavir; ATV, atazanavir; D4T, stavudine; EFV, efavirenz; LPV, lopinavir; NFV, nelfinavir; NVP, nevirapine; TDF, tenofovir; ZDV, zidovudine.

**Figure 2:**
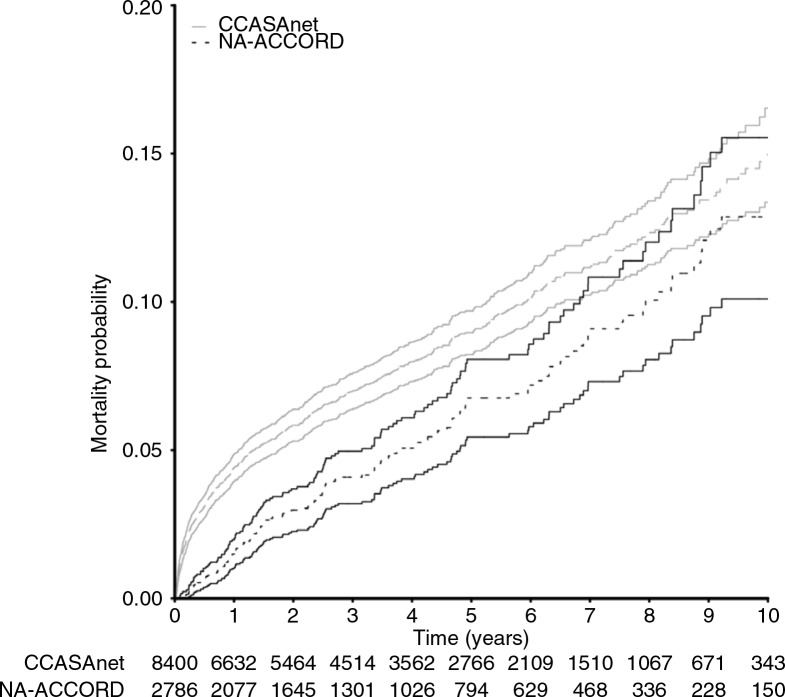
Cumulative mortality among Latino patients initiating ART at Latin American (CCASAnet) and North American (NA-ACCORD) sites. Time zero represents start of first antiretroviral therapy regimen. Solid lines indicate 95% confidence intervals around estimated incidence of mortality.

Crude mortality rates were 2.1 and 1.4 per 100 person-years for ART initiators in CCASAnet and NA-ACCORD, respectively. All-cause mortality early after ART initiation was higher in CCASAnet: 4.4% (95% CI: 4.0 to 4.9%) died during the first year in CCASAnet compared with 1.5% (95% CI: 1.0% to 2.0%) in NA-ACCORD (Figure 2). After one year, the crude mortality rates were 1.28 and 1.33 per 100 person-years for ART initiators who survived the first year in CCASAnet and NA-ACCORD, respectively.

In multivariable Cox analyses, CCASAnet patients had a higher hazard of mortality after ART initiation (adjusted hazard ratio (AHR) 1.61; 95% CI: 1.32 to 1.96), adjusting for age, sex, CD4 count, year of ART initiation, intravenous drug use, prior AIDS diagnosis and ART regimen (Table 2). Among patients who died in the first year, there was no significant difference in CD4 count at ART initiation between CCASAnet and NA-ACCORD (50 vs. 36 cells/µL, *p*=0.35), though CCASAnet patients were younger (37 vs. 42 years, *p*=0.02) and less likely to have an AIDS diagnosis before ART initiation (44% vs. 58%, *p*=0.04; Supplementary Table 1).

**Table 2:** Comparison of all-cause mortality, treatment interruption, regimen change and virologic failure between Latinos initiating antiretroviral therapy in NA-ACCORD and CCASAnet

	Death hazard ratio	Treatment interruption hazard ratio	Second-line regimen switch hazard ratio	Virologic failure hazard ratio
Cohort				
NA-ACCORD	Ref	Ref	Ref	Ref
CCASAnet	1.61 (1.32 to 1.96)	0.46 (0.42 to 0.50)	0.56 (0.51 to 0.62)	0.52 (0.48 to 0.57)
Sex				
Female	Ref	Ref	Ref	Ref
Male	1.02 (0.87 to 1.20)	0.75 (0.68 to 0.82)	0.76 (0.69 to 0.82)	0.75 (0.68 to 0.82)
Age				
20	0.73 (0.52 to 1.04)	1.47 (1.26 to 1.73)	1.25 (1.06 to 1.47)	1.53(1.30 to 1.80)
30	0.82 (0.71 to 0.95)	1.22 (1.12 to 1.34)	1.09 (1.00 to 1.20)	1.04 (0.95 to 1.14)
40	Ref	Ref	Ref	Ref
50	1.31 (1.20 to 1.43)	0.82 (0.76 to 0.88)	0.98 (0.92 to 1.04)	0.93 (0.87 to 0.99)
60	1.77 (1.42 to 2.20)	0.68 (0.55 to 0.83)	0.99 (0.83 to 1.17)	0.78 (0.65 to 0.94)
Clinical AIDS at baseline				
No	Ref	Ref	Ref	Ref
Yes	1.27 (1.07 to 1.49)	1.05 (0.94 to 1.17)	1.04 (0.94 to 1.15)	0.94 (0.85 to 1.05)
Baseline CD4 count, cells/µL				
50	2.28 (1.80 to 2.88)	0.88 (0.78 to 0.99)	1.32 (1.17 to 1.49)	1.05 (0.93 to 1.18)
100	1.68 (1.34 to 2.10)	0.85 (0.76 to 0.95)	1.22 (1.08 to 1.36)	1.00 (0.90 to 1.13)
200	1.09 (0.93 to 1.28)	0.90 (0.84 to 0.95)	1.05 (0.98 to 1.13)	0.90 (0.85 to 0.97)
350	Ref	Ref		Ref
Initiation year				
2000	0.82 (0.62 to 1.09)	1.33 (1.16 to 1.54)	0.86 (0.74 to 1.01)	1.88 (1.62 to 2.18)
2002	0.92 (0.79 to 1.07)	1.25 (1.15 to 1.35)	0.86 (0.79 to 0.94)	1.56 (1.44 to 1.70)
2004	1.00 (0.90 to 1.11)	1.15 (1.08 to 1.22)	0.89 (0.84 to 0.94)	1.27 (1.20 to 1.36)
2006	Ref	Ref	Ref	Ref
2008	0.97 (0.85 to 1.10)	0.85 (0.79 to 0.91)	1.29 (1.20 to 1.38)	0.85 (0.79 to 0.92)
2010	1.02 (0.82 to 1.27)	0.74 (0.65 to 0.84)	1.93 (1.71 to 2.17)	0.95 (0.83 to 1.10)
ART regimen class				
NNRTI	Ref	Ref	Ref	Ref
PI	0.93 (0.77 to 1.13)	1.18 (1.06 to 1.31)	1.05 (0.95 to 1.17)	1.38 (1.24 to 1.53)
Other	1.27 (1.00 to 1.60)	1.50 (1.34 to 1.69)	1.73 (1.53 to 1.94)	1.70 (1.50 to 1.91)
History of IDU				
No	Ref	Ref	Ref	Ref
Yes	1.64 (1.21 to 2.21)	1.42 (1.22 to 1.65)	1.28 (1.08 to 1.51)	1.71 (1.46 to 2.01)

Estimates presented as hazard ratio (95% confidence interval). CCASAnet, Caribbean, Central and South America Network for HIV; IDU, injection drug use; NA-ACCORD, North American AIDS Cohort Collaboration on Research and Design; NNRTI, non-nucleoside reverse transcriptase inhibitor; Ref, reference category; PI, protease inhibitor. Continuous variables were modelled using restricted cubic splines, and the hazard ratios and confidence intervals shown for age, baseline CD4 count, and year of ART initiation represent predicted values estimated from the model.

**Figure 3:**
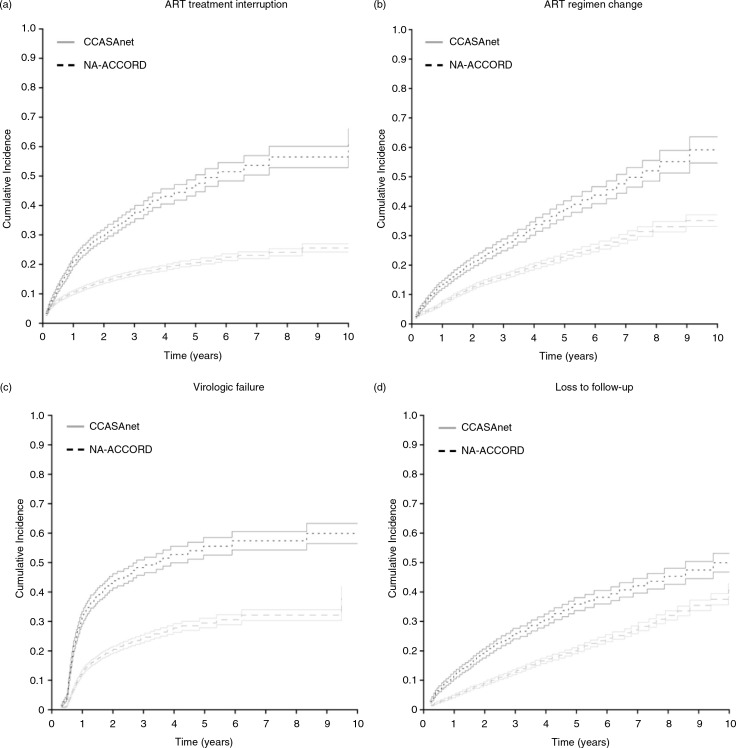
Cumulative incidence of antiretroviral therapy (ART) treatment interruption, ART regimen change, virologic failure and loss to follow-up among CCASAnet and Latino NA-ACCORD patients. All panels show the cumulative incidence of each outcome among CCASAnet and NA-ACCORD patients using a competing risks model where time zero represents the start of the first ART regimen. Solid lines indicate 95% confidence intervals around estimated incidence of treatment interruption. (a) Cumulative incidence of a 14-day or greater ART treatment interruption; (b) cumulative incidence of a change from the initial treatment to a second-line regimen; (c) cumulative incidence of virologic failure among patients who had at least one viral load measurement after treatment initiation; (d) cumulative incidence of loss to follow-up.

The cumulative incidence of an ART treatment interruption, major treatment change, virologic failure and LFU were lower for CCASAnet participants (Figure 3). The cumulative incidence of an ART treatment interruption ≥14 days was lower in CCASAnet compared to NA-ACCORD at one year (0.10 vs. 0.21) and five years (0.21 vs. 0.46). After controlling for characteristics at ART initiation, the hazard ratio for treatment interruption was significantly lower for patients in CCASAnet (AHR: 0.46; 95% CI: 0.42 to 0.50; Table 2).

One and five years after ART initiation, the cumulative incidence of a regimen change among patients initiating ART in CCASAnet was 0.07 and 0.23, respectively, and among Latinos initiating ART in NA-ACCORD, 0.13 and 0.39 (Figure 3b). Similarly, the hazard ratio for changing the initial regimen was significantly lower for patients in CCASAnet (AHR: 0.56; 95% CI: 0.51 to 0.62; Table 2). In CCASAnet, 70% changed from an NNRTI to boosted PI, 24% from one PI to another PI and 2% to a third-line drug. In NA-ACCORD, 41% changed from an NNRTI to a boosted PI, 36% from one PI to another PI and 21% changed to a regimen containing a third-line drug.

Patients initiating ART in CCASAnet also had a lower cumulative incidence of virologic failure (AHR 0.52, 95% CI: 0.48 to 0.57; Table 2 and Figure 3c). The results for virologic failure were not appreciably different when patients were censored at the start of a treatment interruption ≥14 days; the risk of virologic failure remained lower among CCASAnet patients (AHR 0.51, 95% CI: 0.45 to 0.56; data not shown). However, plasma VL was measured less frequently for patients in CCASAnet (median of 1.95 measurements per person per year vs. 3.38 among Latinos in NA-ACCORD). Nonetheless, the percentage of VL measurements after six months that were detectable remained lower for patients in CCASAnet (9.6%; 6157/64,043) than for Latinos in NA-ACCORD (19.7%; 6400/32,423; adjusted relative proportions of 0.53; 95%CI: 0.48 to 0.58).

Lastly, patients initiating ART in CCASAnet had a lower cumulative incidence of LFU than patients initiating ART in NA-ACCORD (Figure 3d). One and five years after ART initiation, the cumulative incidence of LFU among patients initiating ART in CCASAnet was 0.04 and 0.20, respectively, and higher among Latinos initiating ART in NA-ACCORD, 0.12 and 0.36.

### Subregion analyses

Brazilian CCASAnet sites and Brazilian patients enrolled in NA-ACCORD were included in the primary analyses, but given the large component of African migration to Brazil we performed subregion analyses (Supplementary Table 2) that excluded patients initiating ART in Brazil (*n*=1792) and patients born in Brazil initiating ART in North America (*n*=2). The hazard ratio of death in CCASAnet compared to NA-ACCORD was lower when Brazilians were excluded (AHR: 1.27; 95% CI: 1.03 to 1.57), whereas there was little difference in the hazard ratios of treatment interruption, regimen change or virologic failure. Similarly, the percentage of VL measurements that were detectable after six months remained lower for patients initiating ART in CCASAnet in analyses excluding Brazil (adjusted relative proportion=0.52; 95% CI: 0.47 to 0.58).

Among the 804 patients initiating ART in Mexico and 156 Mexicans initiating ART in the United States or Canada, there was insufficient evidence to suggest a difference in mortality (AHR: 1.29; 95% CI: 0.43 to 3.85), although the hazard ratio was in the same direction as primary analyses. Patients initiating ART in Mexico had a lower hazard for virologic failure (AHR: 0.45; 95% CI: 0.33 to 0.63), although again this may have been driven by differences in the frequency of measurements. The percentage of detectable VL measurements was lower among patients initiating ART in Mexico (adjusted relative proportion=0.70; 95% CI: 0.43 to 1.15) but this difference was not statistically significant.

## Discussion

Latinos in the United States and Canada started ART with less severe immunosuppression and had greater survival, particularly in the first 12 months of care, as compared with individuals starting ART in Latin America, but Latinos in North America also appear to be at greater risk for treatment interruptions, treatment regimen changes, virologic failure and LFU.

The lower risk of mortality observed among Latinos in North America compared to those living in Latin America was most pronounced in the early years of ART. Early mortality in resource-limited settings has been well documented, and our results are largely consistent with other comparisons between resource-limited settings (primarily sub-Saharan Africa) and North America and Europe [21]. Patients initiating ART in Latin America had a greater degree of immunosuppression. Although cause of death was not available, other studies have reported AIDS-related conditions as the leading cause of early mortality after treatment initiation in Latin America, and this predominance has not changed despite the improved survival observed in the era of modern combination ART regimens [22–25].

Compared to non-Hispanic whites, Latinos in the United States as a group are diagnosed at a more advanced HIV disease, and foreign-born, non-English speaking and male Latinos are at particularly high risk of delayed diagnosis [26–32]. Barriers to engagement in HIV care in the United States may be greater for Latinos than other ethnic groups. An analysis of 2010 data found that among Hispanics or Latinos diagnosed with HIV, rates at all stages of the care continuum were lower than among non-Hispanic whites [3]. A recent study of a predominantly Hispanic HIV cohort in Texas, found that 46% of patients lacked insurance and an additional 14% were receiving Medicaid [5]. There can also be particular challenges for Latinos due to linguistic barriers and differences in patient-provider communication. For example, prior studies have identified less patient-centred and psychosocial language use by HIV clinicians treating Hispanics versus non-Hispanics [6–8].

Prior studies have also identified important differences among native and foreign-born Latinos in the United States, which may be representative of broader geographic trends. Studies from the United States found that foreign-born Latino males are more likely to have acquired HIV through heterosexual contact as compared to US-born Latinos. Among HIV-positive Latinas, foreign-born women were older at first sexual contact, had fewer episodes of non-HIV sexually transmitted diseases and lower rates of drug and alcohol abuse [27,33,34].

We examined health outcomes at CCASAnet sites in aggregate, but prior studies have found that mortality and programme retention vary considerably between countries in Latin America, which likely reflects differences in the underlying demographics of the HIV epidemic in the region, as well as local social and economic characteristics [9,11–13,35,36]. Heterogeneity in mortality could be related to different programme retention and death ascertainment. Rates of treatment change also vary by CCASAnet sites, with geographic differences in specific toxicities as well as uneven availability of second- and third-line medications, which may be a source of possible confounding [37,38]. The cumulative incidence of regimen change in NA-ACCORD was higher than in CCASAnet, and 21% of patients changing regimens switched to a third-line agent in NA-ACCORD, compared to 2% in CCASAnet. This figure may reflect a greater availability of third-line agents driving a higher cumulative incidence of regimen change, particularly if the change occurs for tolerability issues (which might not be an option in CCASAnet countries with a more limited ART selection). However, it should be noted that cumulative incidence of virologic failure was also higher in NA-ACCORD, so the greater utilization of third-line agents may simply reflect a greater burden of HIV drug resistance.

A strength of this study was the large number of subjects contributing data from multiple countries. However, the largest number of North American Latino patients with known foreign birth location had migrated from Mexico, whereas the majority of CCASAnet patients were located in South America. Limitations of our data included the proportion of patients with no reported country of birth in NA-ACCORD. Other limitations included the lack of information on causes of death and potential confounding of the treatment interruption analysis related to how accurately sites record gaps in ART use (i.e. adherence) versus gaps in prescriptions for ART. Although we assessed time to virologic failure, we did not assess the role of adherence and viral resistance in treatment failure, nor toxicity or other reasons for ART change.

## Conclusions

Mortality rates were higher in Latin America, possibly due to treatment initiation at more advanced stages of HIV disease, whereas treatment interruption, major regimen modification, virologic failure and LFU were higher in North America, where barriers to receiving quality HIV care may be higher for Latino populations. These results emphasize once again the need for early diagnosis and treatment initiation in Latin America and reinforce the key role of ensuring US Latino HIV patients have consistent access to care to improve treatment outcomes.

## Supplementary Material

Health outcomes among HIV-positive Latinos initiating antiretroviral therapy in North America versus Central and South America

## Author information

NA-ACCORD collaborating cohorts and representatives

AIDS Link to the IntraVenous Experience: Gregory D Kirk

Adult AIDS Clinical Trials Group Longitudinal Linked Randomized Trials: Constance A Benson and Ronald J Bosch

Fenway Health HIV Cohort: Stephen Boswell, Chris Grasso and Kenneth H Mayer

HAART Observational Medical Evaluation and Research: Robert S Hogg, P Richard Harrigan, Julio SG Montaner, Angela Cescon and Hasina Samji

HIV Outpatient Study: John T Brooks and Kate Buchacz

HIV Research Network: Kelly A Gebo and Richard D Moore

Johns Hopkins HIV Clinical Cohort: Richard D Moore

John T. Carey Special Immunology Unit Patient Care and Research Database, Case Western Reserve University: Benigno Rodriguez

Kaiser Permanente Mid-Atlantic States: Michael A Horberg

Kaiser Permanente Northern California: Michael J Silverberg

Longitudinal Study of Ocular Complications of AIDS: Jennifer E Thorne

Multicenter Hemophilia Cohort Study–II: James J Goedert

Multicenter AIDS Cohort Study: Lisa P Jacobson and Gypsyamber D'Souza

Montreal Chest Institute Immunodeficiency Service Cohort: Marina B Klein

Ontario HIV Treatment Network Cohort Study: Sean B Rourke, Ann N Burchell and Anita R Rachlis

Retrovirus Research Center, Bayamon Puerto Rico: Robert F Hunter-Mellado and Angel M Mayor

Southern Alberta Clinic Cohort: M John Gill

Studies of the Consequences of the Protease Inhibitor Era: Steven G Deeks and Jeffrey N Martin

The Study to Understand the Natural History of HIV/AIDS in the Era of Effective Therapy: Pragna Patel and John T Brooks

University of Alabama at Birmingham 1917 Clinic Cohort: Michael S Saag, Michael J Mugavero and James Willig

University of North Carolina at Chapel Hill HIV Clinic Cohort: Joseph J Eron and Sonia Napravnik

University of Washington HIV Cohort: Mari M Kitahata, Heidi M Crane and Daniel R Drozd

Veterans Aging Cohort Study: Amy C Justice, Robert Dubrow and David Fiellin

Vanderbilt Comprehensive Care Clinic HIV Cohort: Timothy R Sterling, David Haas, Peter Rebeiro, Sally Bebawy and Megan Turner

Women's Interagency HIV Study: Stephen J Gange and Kathryn Anastos

NA-ACCORD study administration

Executive Committee: Richard D Moore, Michael S Saag, Stephen J Gange, Mari M Kitahata, Keri N Althoff, Rosemary G McKaig, Amy C Justice and Aimee M Freeman

Administrative Core: Richard D Moore, Aimee M Freeman and Carol Lent

Data Management Core: Mari M Kitahata, Stephen E Van Rompaey, Heidi M Crane, Daniel R Drozd, Liz Morton, Justin McReynolds and William B Lober

Epidemiology and Biostatistics Core: Stephen J Gange, Keri N Althoff, Alison G Abraham, Bryan Lau, Jinbing Zhang, Yuezhou Jing, Elizabeth Golub, Shari Modur, Cherise Wong, Bin Liu, Weiqun Tong and Brenna Hogan

CCASAnet cohorts and representatives

Fundación Huésped, Buenos Aires, Argentina, principal investigator (PI): Pedro Cahn, MD, PhD

Instituto de Pesquisa Clinica Evandro Chagas, Fundação Oswaldo Cruz, Rio de Janeiro, Brazil, PI: Beatriz Grinsztejn, MD, PhD

Universidad de Chile, Santiago, Chile, PI: Marcelo Wolff Reyes, MD

Le Groupe Haïtien d'Etude du Sarcome de Kaposi et des Infections Opportunistes, Port-au-Prince, Haiti, PI: Jean W Pape, MD

Instituto Hondureño de Seguridad Social and Hospital Escuela, Tegucigalpa, Honduras, PI: Denis Padgett, MD

Instituto Nacional de Ciencias Médicas y Nutrición, Mexico City, Mexico, PI: Juan Sierra Madero, MD

Instituto de Medicina Tropical Alexander von Humboldt, Lima, Peru, PI: Eduardo Gotuzzo, MD

Vanderbilt University, Nashville, TN, USA, PI: Catherine McGowan, MD

CCASAnet PIs

Pedro Cahn (pedro.cahn@huesped.org.ar)

Catherine McGowan (c.mcgowan@Vanderbilt.Edu)

## References

[1] UNAIDS (2013). Global report: UNAIDS report on the global AIDS epidemic 2013.

[2] Ennis SR, Rios-Vargas M, Albert NG (2011). The Hispanic population: 2010.

[3] Gant Z, Bradley H, Hu X, Skarbinski J, Hall HI, Lansky A (2014). Hispanics or Latinos living with diagnosed HIV: progress along the continuum of HIV care – United States, 2010. MMWR Morb Mortal Wkly Rep.

[4] Centers for Disease Control and Prevention (2013). HIV surveillance report, 2011.

[5] Taylor BS, Liang Y, Garduno LS, Walter EA, Gerardi MB, Anstead GM (2014). High risk of obesity and weight gain for HIV-infected uninsured minorities. J Acquir Immune Defic Syndr.

[6] Garland JM, Andrade AS, Page KR (2010). Unique aspects of the care of HIV-positive Latino patients living in the United States. Curr HIV/AIDS Rep.

[7] Beach MC, Saha S, Korthuis PT, Sharp V, Cohn J, Wilson I (2010). Differences in patient-provider communication for Hispanic compared to non-Hispanic white patients in HIV care. J Gen Intern Med.

[8] Chen NE, Gallant JE, Page KR (2012). A systematic review of HIV/AIDS survival and delayed diagnosis among Hispanics in the United States. J Immigr Minor Health.

[9] Tuboi SH, Schechter M, McGowan CC, Cesar C, Krolewiecki A, Cahn P (2009). Mortality during the first year of potent antiretroviral therapy in HIV-1-infected patients in 7 sites throughout Latin America and the Caribbean. J Acquir Immune Defic Syndr.

[10] Brinkhof MW, Dabis F, Myer L, Bangsberg DR, Boulle A, Nash D (2008). Early loss of HIV-infected patients on potent antiretroviral therapy programmes in lower-income countries. Bull World Health Organ.

[11] Wolff MJ, Cortes CP, Shepherd BE, Beltran CJ, Chilean ACSG (2010). Long-term outcomes of a national expanded access program to antiretroviral therapy: the Chilean AIDS cohort. J Acquir Immune Defic Syndr.

[12] Gupta A, Nadkarni G, Yang WT, Chandrasekhar A, Gupte N, Bisson GP (2011). Early mortality in adults initiating antiretroviral therapy (ART) in low – and middle-income countries (LMIC): a systematic review and meta-analysis. PLoS One.

[13] Carriquiry G, Fink V, Koethe J, Giganti M, Jayathilake K, Cahn P (2015). Mortality and loss to follow-up among HIV-infected persons on long-term antiretroviral therapy in Latin America and the Caribbean. J Int AIDS Soc.

[14] Braitstein P, Brinkhof MW, Dabis F, Schechter M, Boulle A, Miotti P (2006). Mortality of HIV-1-infected patients in the first year of antiretroviral therapy: comparison between low-income and high-income countries. Lancet.

[15] Sanne IM, Westreich D, Macphail AP, Rubel D, Majuba P, Van Rie A (2009). Long term outcomes of antiretroviral therapy in a large HIV/AIDS care clinic in urban South Africa: a prospective cohort study. J Int AIDS Soc.

[16] Sterne JA, May M, Costagliola D, de Wolf F, Phillips AN, Harris R (2009). Timing of initiation of antiretroviral therapy in AIDS-free HIV-1-infected patients: a collaborative analysis of 18 HIV cohort studies. Lancet.

[17] McGowan CC, Cahn P, Gotuzzo E, Padgett D, Pape JW, Wolff M (2007). Cohort Profile: Caribbean, Central and South America Network for HIV research (CCASAnet) collaboration with in the International Epidemiologic Databases to Evaluate AIDS (IeDEA) programme. Int J Epidemiol.

[18] Gange SJ, Kitahata MM, Saag MS, Bangsberg DR, Bosch RJ, Brooks JT (2007). Cohort profile: the North American AIDS Cohort Collaboration on Research and Design (NA-ACCORD). Int J Epidemiol.

[19] Harrell FE (2001). Regression modeling strategies with applications to linear models, logistic regression, and survival analysis.

[20] Rubin DB, Schenker N (1991). Multiple imputation in health-care databases: an overview and some applications. Stat Med.

[21] Boulle A, Schomaker M, May MT, Hogg RS, Shepherd BE, Monge S (2014). Mortality in patients with HIV-1 infection starting antiretroviral therapy in South Africa, Europe, or North America: a collaborative analysis of prospective studies. PLoS Med.

[22] Perez E, Toibaro JJ, Losso MH (2005). [HIV patient hospitalization during the pre and post-HAART era]. Medicina (B Aires).

[23] Pacheco AG, Tuboi SH, May SB, Moreira LF, Ramadas L, Nunes EP (2009). Temporal changes in causes of death among HIV-infected patients in the HAART era in Rio de Janeiro, Brazil. J Acquir Immune Defic Syndr.

[24] Grinsztejn B, Luz PM, Pacheco AG, Santos DV, Velasque L, Moreira RI (2013). Changing mortality profile among HIV-infected patients in Rio de Janeiro, Brazil: shifting from AIDS to non-AIDS related conditions in the HAART era. PLoS One.

[25] Grinsztejn B, Veloso VG, Friedman RK, Moreira RI, Luz PM, Campos DP (2009). Early mortality and cause of deaths in patients using HAART in Brazil and the United States. AIDS.

[26] Espinoza L, Hall HI, Hu X (2009). Increases in HIV diagnoses at the U.S.-Mexico border, 2003–2006. AIDS Educ Prev.

[27] Espinoza L, Hall HI, Selik RM, Hu X (2008). Characteristics of HIV infection among Hispanics, United States 2003–2006. J Acquir Immune Defic Syndr.

[28] Hall HI, Byers RH, Ling Q, Espinoza L (2007). Racial/ethnic and age disparities in HIV prevalence and disease progression among men who have sex with men in the United States. Am J Public Health.

[29] Hall HI, Geduld J, Boulos D, Rhodes P, An Q, Mastro TD (2009). Epidemiology of HIV in the United States and Canada: current status and ongoing challenges. J Acquir Immune Defic Syndr.

[30] Hall HI, McDavid K, Ling Q, Sloggett A (2006). Determinants of progression to AIDS or death after HIV diagnosis, United States, 1996 to 2001. Ann Epidemiol.

[31] Wohl AR, Tejero J, Frye DM (2009). Factors associated with late HIV testing for Latinos diagnosed with AIDS in Los Angeles. AIDS Care.

[32] Torrone EA, Thomas JC, Leone PA, Hightow-Weidman LB (2007). Late diagnosis of HIV in young men in North Carolina. Sex Transm Dis.

[33] Prosser AT, Tang T, Hall HI (2012). HIV in persons born outside the United States, 2007–2010. JAMA.

[34] Castillo-Mancilla J, Allshouse A, Collins C, Hastings-Tolsma M, Campbell TB, Mawhinney S (2012). Differences in sexual risk behavior and HIV/AIDS risk factors among foreign-born and US-born Hispanic women. J Immigr Minor Health.

[35] Yiannoutsos CT, Johnson LF, Boulle A, Musick BS, Gsponer T, Balestre E (2010). Estimated mortality of adult HIV-infected patients starting treatment with combination antiretroviral therapy. Sex Transm Infect.

[36] World Bank World bank open data 2014.

[37] Cesar C, Shepherd BE, Krolewiecki AJ, Fink VI, Schechter M, Tuboi SH (2010). Rates and reasons for early change of first HAART in HIV-1-infected patients in 7 sites throughout the Caribbean and Latin America. PLoS One.

[38] Cesar C, Shepherd BE, Jenkins CA, Ghidinelli M, Castro JL, Veloso VG (2014). Use of third line antiretroviral therapy in Latin America. PLoS One.

