# Supplementary material for: Health outcomes among HIV-positive Latinos initiating antiretroviral therapy in North America versus Central and South America
Source: J Int AIDS Soc. 2016 Mar 18;19(1):20684. doi: 10.7448/IAS.19.1.20684 (PMC4800379; doi:10.7448/IAS.19.1.20684)
Supplement: Health outcomes among HIV-positive Latinos initiating antiretroviral therapy in North America versus Central and South America [file JIAS-19-20684-s001.pdf]

Supplementary Tables and Figures

Supplementary Table 1. Demographic and clinical characteristics of patients deceased in the first year of antiretroviral therapy

	CCASAnet (n=347)	NA-ACCORD (n=36)	p-value
Proportion of total cohort, %	4.1%	1.3%	<0.01
Age, years	37 (31 - 45)	42 (35 - 47.5)	0.02
Sex			
Female	85 (24.5%)	9 (25.0%)	0.99
Male	262 (75.5%)	27 (75.0%)	
Route of infection			
Heterosexual	185 (53.3%)	12 (33.3%)	< 0.001
MSM	92 (26.5%)	11 (30.6%)	
IDU	7 (2.0%)	9 (25.0%)	
Other	6 (1.7%)	3 (8.3%)	
Unknown	57 (16.4%)	1 (2.8%)	
Clinical stage			0.04
AIDS	151 (43.5%)	21 (58.3%)	
not AIDS	150 (43.2%)	8 (22.2%)	
Missing	46 (13.3%)	7 (19.4%)	
Nadir CD4 count, cells/µL	51 (16 - 120)	36 (5 - 105)	0.15
Missing	68 (20%)	1(3%)	
Baseline CD4 count, cells/µL	50 (16 - 114)	36 (8 - 111)	0.35
Missing	79 (23%)	3 (8%)	
Baseline viral load (log_{10})	5.1 (4.7 - 5.7)	5.3 (4.7 - 5.6)	0.97
Initial regimen			< 0.001
NNRTI	280(80.7%)	7(19.4%)	
Boosted PI	49(14.1%)	16(44.4%)	
Other	18(5.2%)	13(36.1%)	
Initiation Year	2006 (2004 - 2009)	2004 (2002 - 2006)	< 0.001
Viral load measurements per person-year	0.00 (0.00 - 1.72)	4.31 (1.90 - 6.51)	< 0.001
At least one VL measurement per year of follow-up			< 0.001
Yes	106(30.5%)	31(86.1%)	
No	241(69.5%)	5(13.9%)	

Abbreviations: IDU, injection drug use. MSM, men who have sex with men; NNRTI, non-nucleoside reverse transcriptase inhibitor, PI, protease inhibitor.

Supplementary Table 2. Subregion-specific mortality, treatment interruption, regimen change, and virologic failure for CCASAnet patients compared to Latinos in NA-ACCORD*

Geographic region	Death Hazard Ratio	Treatment Interruption Hazard Ratio	Second-line Regimen Switch Hazard Ratio	Virologic Failure Hazard Ratio
All Latin America	1.61 (1.32 - 1.96)	0.46 (0.42 - 0.50)	0.56 (0.51 – 0.62)	0.52 (0.48 - 0.57)
Latin America without Brazil	1.27 (1.03 -1.57)	0.47 (0.43 – 0.52)	0.46 (0.42 – 0.51)	0.50 (0.45 – 0.55)
Mexico only	1.29 (0.43-3.85)	0.06 (0.03-0.11)	0.66 (0.46 – 0.96)	0.45 (0.33-0.63)

*Latinos in NA-ACCORD are the reference group for all comparisons

Estimates presented as Hazard Ratio (95% Confidence Interval)
